# Case Report: CD19 and CD22 CAR-T therapy induced durable complete remission in a patient with refractory plasmablastic lymphoma

**DOI:** 10.3389/fimmu.2025.1697566

**Published:** 2025-10-29

**Authors:** Dan Liu, Haichen Wei, Yanran Hu, Hui Wang, Ping Li, Ke Lu, Wenjun Zhang, Lijie Xing, Zengjun Li

**Affiliations:** ^1^ Department of Lymphoma and Hematology, Shandong Cancer Hospital and Institute, Shandong First Medical University and Shandong Academy of Medical Sciences, Jinan, China; ^2^ Pathology Department, Shandong Cancer Hospital and Institute, Shandong First Medical University and Shandong Academy of Medical Sciences, Jinan, China

**Keywords:** plasmablastic lymphoma, refractory, car-t, bortezomib, daratumumab

## Abstract

**Introduction:**

Plasmablastic lymphoma (PBL) is a rare and highly aggressive form of non-Hodgkin lymphoma that is associated with a poor prognosis. Traditional chemotherapy has demonstrated limited efficacy for PBL. There is currently no standard treatment for patients with refractory or relapsing PBL. While CAR-T therapy has shown promising outcomes in leukemia, lymphoma and myeloma, evidence of its application in PBL remains scarce.

**Case presentation:**

We describe a 56-year-old patient diagnosed with PBL. The patient achieved short-term remission with bortezomib in combination with etoposide, dexamethasone, cyclophosphamide, and doxorubicin as first-line therapy. After disease progression, the patient received daratumumab combined with GemOx as second-line treatment but showed no response. Tumor biopsy after disease progression revealed strong positive CD22 and partial positive CD19 expression. The patient received CD19 and CD22 CAR-T therapies as the third-line treatment and achieved durable complete remission for more than one year with good tolerance.

**Conclusion:**

A patient with refractory PBL achieved durable complete remission after CD19 and CD22 CAR-T therapies, suggesting this treatment may be effective for patients with refractory or relapsing PBL.

## Introduction

Plasmablastic lymphoma (PBL) is a rare and aggressive form of non-Hodgkin lymphoma that is CD20-negative, representing less than 1% of all diffuse large B cell lymphoma (DLBCL) (1). PBL is primarily found in HIV-positive individuals, with 50%–85% of cases associated with HIV (2–4). Chronic Epstein–Barr virus infection and MYC gene rearrangements also contribute to the development of PBL (2, 4). PBL typically involves extra-nodal sites, including the oral cavity and gastrointestinal tract (2, 5). PBL is histologically characterized by the proliferation of plasmablasts or immunoblasts with a high proliferation index (2). The immunophenotype of PBL is characterized by positivity for plasma cell markers such as CD38, CD138, MUM1, and CD79a and negativity for B cell markers such as CD20 and PAX-5 (5, 6).

The prognosis is poor for patients with PBL treated with traditional therapies, such as cyclophosphamide, doxorubicin, vincristine and prednisone (CHOP) or dose-adjusted infusional etoposide, vincristine, and doxorubicin with bolus cyclophosphamide and prednisone (DA-EDOCH) (4). The median overall survival of patients with PBL ranges from 1 to 2 years (7, 8). The combination of anti-myeloma agents such as bortezomib, lenalidomide, and daratumumab has led to better treatment outcomes compared with chemotherapy alone (5, 9).

Chimeric antigen receptor T cell (CAR-T) therapy has shown encouraging responses in patients with B cell lymphoma and leukemia. However, reports on CAR-T therapy for PBL are very limited. In this case report, we describe a patient with refractory PBL who received CD19 and CD22 CAR-T therapies and achieved sustained complete remission (CR).

## Case description

**Figure 1:**
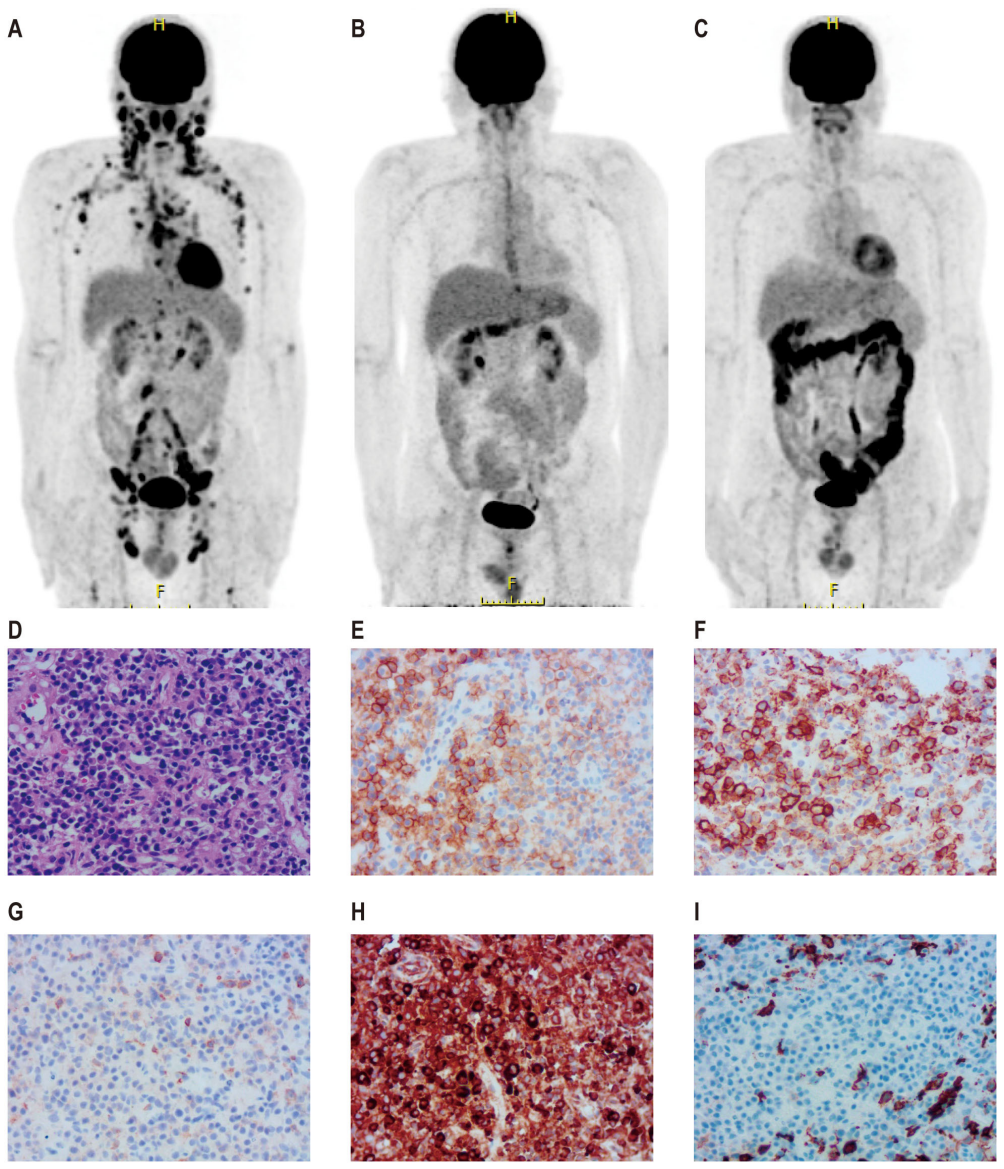
PET-CT images and biopsy of lymph node of a patient with plasmablastic lymphoma. Pre-treatment PET-CT **(A)**, PET-CT after four cycles of first-line therapy **(B)**, and PET-CT three months after CD19 and CD22 CAR-T treatment **(C)**. A biopsy of the right pelvic mass was conducted prior to CAR-T therapy. Wright–Giemsa staining revealed abnormal cells characterized by diffuse proliferation and infiltration, large size, abundant cytoplasm, round or slightly irregular nuclei, prominent central nucleoli, and frequent mitotic figures **(D)**. Immunohistochemical analysis demonstrated that tumor cells were positive for CD38 **(E)**, CD138 **(F)**, CD19 **(G)**, and CD22 **(H)** and negative for CD20 **(I)**.

A 56-year-old male presented to a local hospital with a two-month history of right neck lymph node enlargement. He underwent a biopsy of the left paracervical lymph node. Biopsy results led to a diagnosis of DLBCL by local pathologists. Flow cytometry analysis of bone marrow puncture revealed monoclonal B lymphocytes expressing CD19, CD20, CD11c, and Ki-67 and lacking CD5, CD10, CD25, CD38, CD103, and CD123 expression. The patient was admitted to our hospital for treatment. Pathologists in our hospital reviewed the pathological section and made a diagnosis of PBL. Immunohistochemistry showed positive CD38, CD138, CD19, CD22, CD79a, Mum1, and kappa chain; negative CD3, CD5, CD10, CD20, PAX-5, BCL2, BCL6, C-myc, CD56, cyclin D1, ALKp80, CK, EBER, EMA, and lambda chain; 10% CD30 positivity; FDC network-positive staining for CD21; and a Ki-67 positivity rate of 60%. PET/CT examination showed increased radiation uptake in the nasopharyngeal region, tonsils, and many lymph nodes above and below the diaphragm, with a maximum standardized uptake value of 15.5 (**Figure 1A**).

The patient was diagnosed with stage IV PBL and was treated with a regimen consisting of bortezomib in combination with etoposide, dexamethasone, cyclophosphamide, and doxorubicin (EDCH) (bortezomib 2.6 mg d1, 4; etoposide 0.1 g d1–4; dexamethasone 20 mg d1, 2, 4, 5; cyclophosphamide 750 mg/m^2^ d1; doxorubicin liposome 60 mg d1, q3w). The patient achieved CR after four cycles of first-line treatment. The Deauville Score by PET-CT was three (**Figure 1B**). Consolidation with autologous stem cell transplantation was suggested but declined by the patient because of financial constraints. He received bortezomib in combination with the EDCH regimen for two more cycles. At one month after the completion of six cycles of EDCH chemotherapy and bortezomib, CT scan indicated disease progression. Multiple enlarged lymph nodes were observed in several areas above and below the diaphragm. The largest lymph node, measuring approximately 2.9 cm × 1.6 cm, was in the left neck. The spleen was larger than previous scans, indicating possible involvement.

**Figure 2:**
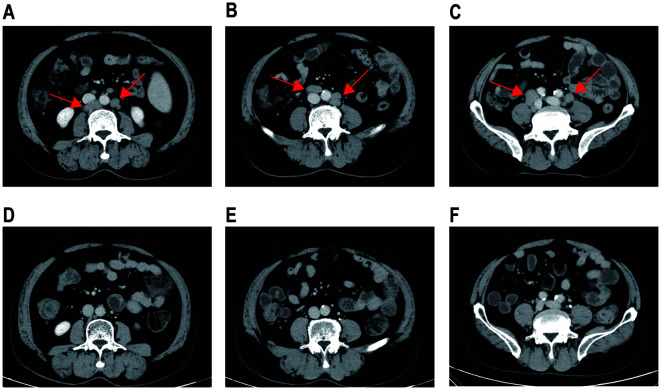
CT images before and after CAR-T therapy. CT scans following two cycles of daratumumab in combination with GemOx showed no treatment response **(A–C)**. One month after the transfusion of CD19 and CD22 CAR-T cells, the patient achieved a complete response **(D–F)**.

The patient received second-line treatment: daratumumab combined with GemOx for two cycles (daretuzumab 800 mg qw; gemcitabine 1 g/m^2^ d1, oxaliplatin 100 mg/m^2^ d1, q2w). CT scans revealed disease progression after two cycles of second-line treatment (**Figures 2A–C**). The largest lymph nodes were in the pelvic cavity, with a cross-section of 4.8 cm × 2.4 cm. A biopsy of the right pelvic mass was performed. The pathological results indicated the presence of PBL. Tumor cells were positive for CD38, CD138, CD22, CD79a, MUM1, CD45, and kappa; partial positive for CD19 and EBER; negative for CD2, CD3, CD5, CD10, CD20, CD56, cyclin D1, ALKp80, HHV-8, MPO, lambda and IgG; and 5% positive for CD30; the Ki-67 positivity rate was 70% (**Figures 1D–I**). Immunostaining revealed CD19 expression in 15% of tumor cells and CD22 expression in nearly all cells (95%). As CD19 and CD22 are targets of CAR-T cell therapy, CAR-T therapy was considered as third-line treatment.

**Figure 3:**
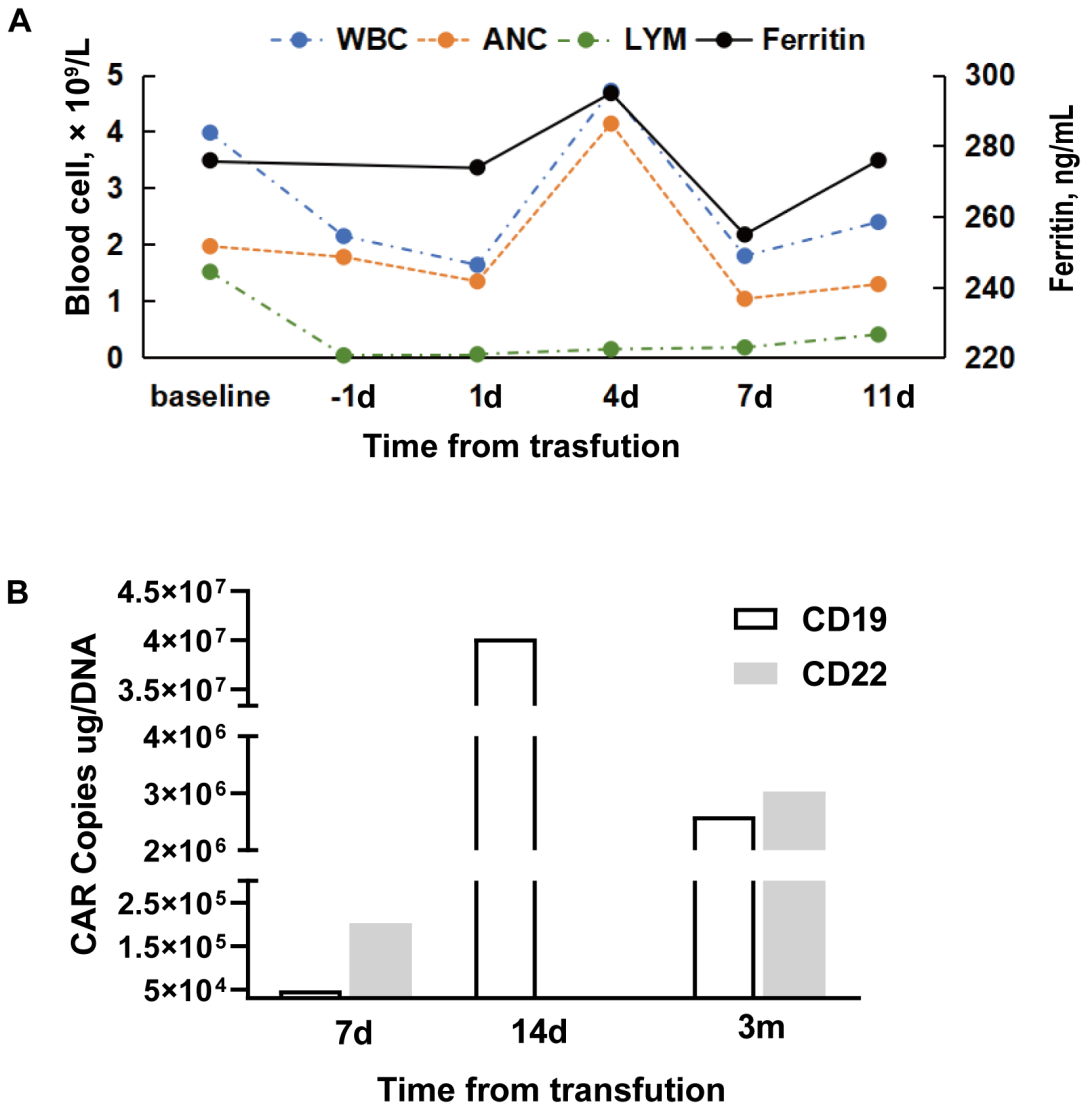
Blood cell counts, ferritin, and CAR copies during CAR-T therapy. **(A)** Ferritin (ng/mL), leukocyte (×10^9^/L), neutrophil (×10^9^/L), and lymphocyte (×10^9^/L) at baseline (prior to chemotherapy of fludarabine and cyclophosphamide) and following CD19 CAR-T cell transfusion. **(B)** CD19 and CD22 CAR copy numbers post-infusion. (CD22 CAR copy data could not be obtained as scheduled on day 14 following infusion because of the patient’s discharge from the hospital). WBC, white blood cell count; ANC, absolute neutrophil count; Lym, lymphocyte count.

Peripheral blood was collected for the generation of two distinct CAR-T cell products targeting CD19 and CD22, respectively. The CD19-directed CAR-T cells feature an anti-CD19 single-chain variable fragment (scFv), fused to a CD8α hinge and transmembrane domain, a 4-1BB (CD137) costimulatory domain, and a CD3ζ intracellular signaling domain. The CD22-specific CAR-T construct followed a similar design, with the anti-CD19 scFv substituted by an anti-CD22 scFv. The patient received cyclophosphamide and fludarabine for lymphodepletion. CD19 CAR-T cells (2.9 × 10^6^/kg) followed by CD22 CAR-T cells (3.7 × 10^6^/kg) were transfused sequentially to the patient with an interval of six days. White blood cells and neutrophilic granulocytes decreased after chemotherapy, but without neutropenia (**Figure 3A**). The patient showed no cytokine release syndrome response after CAR-T therapy. Ferritin slightly increased on day 4 following CD19 CAR-T transfusion (**Figure 3A**). CAR-T cells increased following transfusion and were detectable three months after transfusion (**Figure 3B**). One month after CAR-T transfusion, CT scan showed no enlarged lymph nodes (**Figures 2D–F**). Three months later, PET-CT showed a complete metabolic response (**Figure 1C**). Since the CAR-T transfusion, the duration of complete remission was more than 12 months until this submission.

## Discussion

PBL is a rare type of B cell non-Hodgkin lymphoma, with characteristics of both lymphoma and plasma cell neoplasms (2, 5). Previous studies reported a high association of PBL with immunosuppression, male sex, Epstein–Barr virus infection, oral cavity involvement, and aggressive clinical behavior (10). While the CHOP regimen is an established standard for various non-Hodgkin lymphomas as indicated in NCCN guidelines, it exhibits limited efficacy in patients with PBL. In a meta-analysis including 173 patients with PBL, CHOP was the most common regimen for the first-line treatment of PBL, and the CR rate was 39% (4). PBL shares some biological and phenotypic features with multiple myeloma; therefore, drugs for plasma may be effective for PBL. Lenalidomide/thalidomide-containing regimens and bortezomib-containing regimens led to a high CR rate (100% and 50%, respectively) in patients with PBL (4). Autologous stem cell transplantation, both in the setting of initial therapy and after relapse, may be considered for patients who achieved CR. A review of 24 patients with PBL who received autologous stem cell transplantation post-frontline therapy reported a CR rate of 50% and partial response rate of 17% (11). The two-year relapse rate was 30%. In this case, the patient was treated with bortezomib plus EDCH and achieved a transient response; however, the disease relapsed shortly thereafter.

There is currently no standard treatment for patients with relapsing refractory PBL. Tumor cells of patients with PBL are often positive for CD38; therefore, CD38 antibody is a reasonable treatment option. Dittus et al. reported four patients with relapsed PBL who received daratumumab and ifosfamide, carboplatin, and etoposide (ICE) and achieved CR (12). However, the progression-free survival was short, with a median of 6.5 months. The patient in this case received daratumumab in combination with chemotherapy as second-line treatment, but the disease subsequently progressed.

CAR-T therapy was reported to achieve a remarkable response in patients with B cell lymphomas, myeloma, and leukemia. Patients with PBL also express targets for CAR-T therapy such as BCMA, CD19, and CD22, but reports on CAR-T therapy in patients with PBL are very rare. Previous studies reported three patients with PBL who received CAR-T therapy (13–15). Ruben et al. reported a patient with refractory PBL with no response to EDOCH, ICE, and anti-plasma therapies, including carfilzomib, lenalidomide, and daratumumab (13). The patient finally received CD19 CAR-T and achieved a short-term CR; the disease relapsed five months after CAR-T transfusion. Raghunanda et al. reported a patient with B cell leukemia who relapsed after chemotherapy, CD22 antibody, and CD19 CAR-T therapy; the biopsy revealed PBL (14). The patient achieved partial remission after combined treatment of lenalidomide, daratumumab, and ibrutinib. BCMA CAR-T therapy was administered, and the patient achieved CR. Feng et al. reported a 61-year-old patient with chronic lymphoma leukemia that transformed into PBL after receiving ibrutinib (15). The patient achieved CR after three cycles of cyclophosphamide, doxorubicin, and prednisone (CHP) together with venetoclax and brentuximab vedotin. Immunohistochemistry revealed positive BCMA. The patient received BCMA CAR-T therapy and achieved durable CR for six months (15).

CD19 CAR-T therapy has been widely used in the treatment of B cell lymphoma and leukemia; however, a subset of patients eventually relapses because of antigen loss or downregulation on malignant cells (16). The combination of CD22 CAR-T therapy is a potential strategy to overcome this limitation. In our center, selected patients with lymphoma received sequential transfusions of anti-CD19 and anti-CD22 CAR-T cells when immunohistochemistry confirmed co-expression of both antigens. In these cases, the rates of toxicities such as cytokine release syndrome and neurotoxicity were comparable to those observed in patients receiving CD19 CAR-T therapy alone.

Here we described a patient with PBL who achieved short-term remission with bortezomib in combination with EDCH as first-line therapy but showed no response with daratumumab combined with GemOx as second-line treatment. Tumor biopsy after disease progression revealed strong positive CD22 and partial positive CD19 expression. The patient received CD19 and CD22 CAR-T therapies as the third-line treatment and achieved durable CR for more than one year with good tolerance. There is currently no standard therapy for recurrent and refractory PBL. Thus, this case indicates that CD19 and CD22 CAR-T therapy may be a potential treatment for these patients. However, this report represents a single case study. Further studies in larger patient groups are required to explore the efficacy of CD19 and CD22 CAR-T therapy for this patient group.

## Data Availability

The original contributions presented in the study are included in the article/Supplementary Material. Further inquiries can be directed to the corresponding author.
